# A Promoter in the Coding Region of the Calcium Channel Gene *CACNA1C* Generates the Transcription Factor CCAT

**DOI:** 10.1371/journal.pone.0060526

**Published:** 2013-04-16

**Authors:** Natalia Gomez-Ospina, Georgia Panagiotakos, Thomas Portmann, Sergiu P. Pasca, Dania Rabah, Agata Budzillo, Jean Pierre Kinet, Ricardo E. Dolmetsch

**Affiliations:** 1 Department of Neurobiology, Stanford University School of Medicine, Stanford, California, United States of America; 2 Department of Pathology, Harvard University Medical School and Beth Israel Medical Center, Boston, Massachusetts, United States of America; 3 Biogen Idec Inc, Cambridge, Massachusetts, United States of America; University of California, San Diego, United States of America

## Abstract

The C-terminus of the voltage-gated calcium channel Ca_v_1.2 encodes a transcription factor, the calcium channel associated transcriptional regulator (CCAT), that regulates neurite extension and inhibits Ca_v_1.2 expression. The mechanisms by which CCAT is generated in neurons and myocytes are poorly understood. Here we show that CCAT is produced by activation of a cryptic promoter in exon 46 of *CACNA1C,* the gene that encodes Ca_V_1.2. Expression of CCAT is independent of Ca_v_1.2 expression in neuroblastoma cells, in mice, and in human neurons derived from induced pluripotent stem cells (iPSCs), providing strong evidence that CCAT is not generated by cleavage of Ca_V_1.2. Analysis of the transcriptional start sites in *CACNA1C* and immune-blotting for channel proteins indicate that multiple proteins are generated from the 3′ end of the *CACNA1C* gene. This study provides new insights into the regulation of *CACNA1C,* and provides an example of how exonic promoters contribute to the complexity of mammalian genomes.

## Introduction

Voltage-gated calcium channels are central regulators of signaling and gene expression in the nervous system. The L-type calcium channel Ca_v_1.2 is particularly effective at regulating gene expression in response to depolarization [1], [2]. This transcriptional regulation can be mediated by influencing the function of transcription factors like CREB, NFAT, and MEF2 [3]–[7], or by the activity of CCAT, a transcription factor encoded within its C-terminal domain. We previously reported that CCAT is localized to the nucleus of neurons, has a potent transactivation domain, and regulates the expression of endogenous genes including Connexin 31.1 and NR3 [8]. CCAT has also been reported to repress the expression of Ca_v_1.2, suggesting that it provides negative feedback to regulate channel expression [9].

The mechanisms by which CCAT is generated in neurons and myocytes are not well understood. Biochemical studies have suggested that Ca_v_1.2 is cleaved at its C-terminus in muscle cells to generate a free C-terminal fragment. A putative cleavage site on the C-terminus of Ca_v_1.1, the L-type channel in skeletal muscle, has been identified using mass spectrometry [10]. It is not clear, however, if a similar mechanism is responsible for generating CCAT in neurons.

## Results

### CCAT Expression is Independent of the Ca_v_1.2 Channel Protein

**Figure 1:**
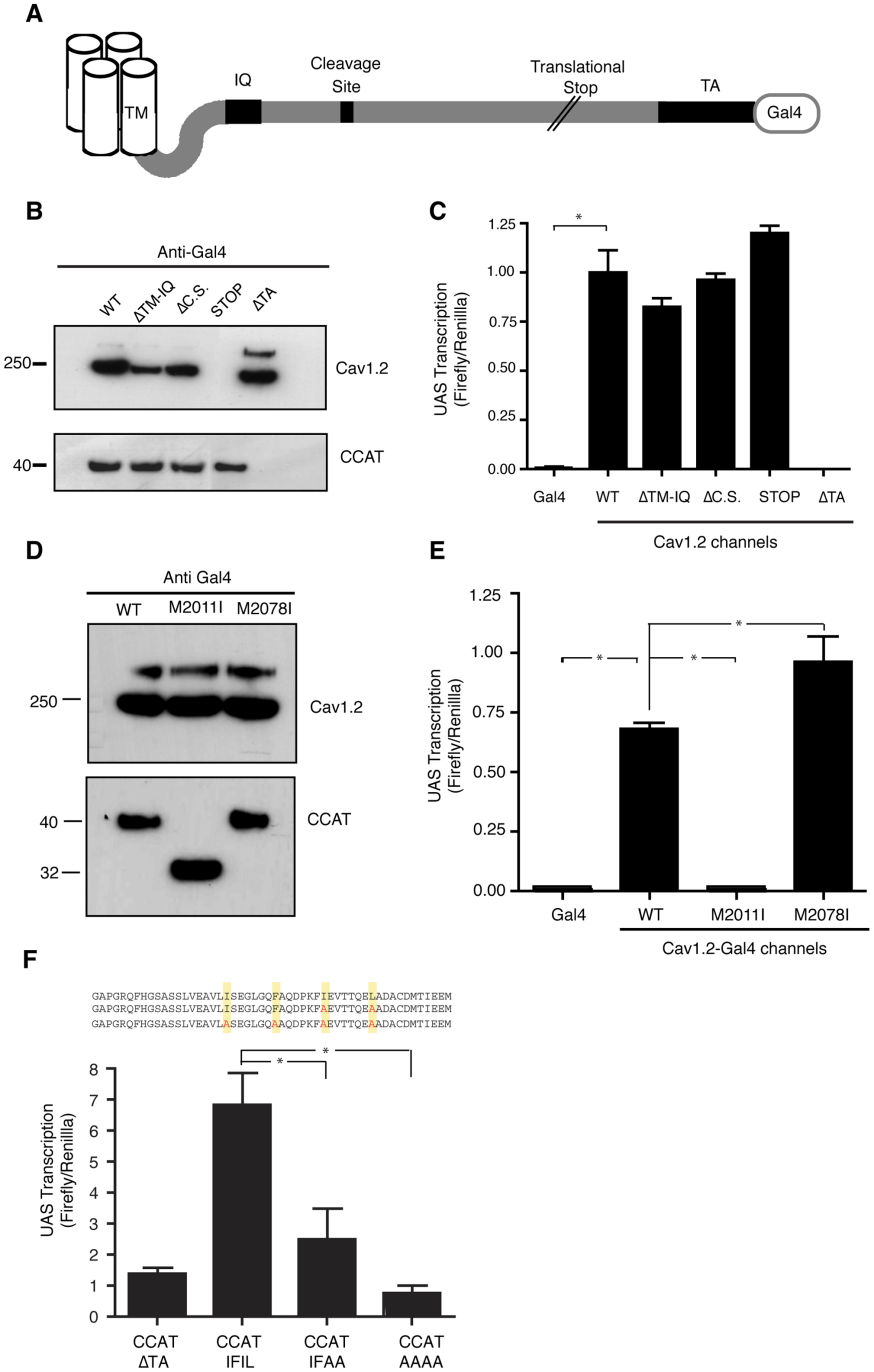
CCAT Expression is Independent of Ca_v_1.2 Channel Protein. (A) Schematic representation of the Ca_v_1.2-Gal4 fusion and channel mutants. Four mutations are depicted: ΔTM-IQ is a deletion from the TM to IQ motif that renders the channel unable to traffic to the membrane. ΔC.S is a deletion of the conserved cleavage site in Ca_v_1.1. STOP is a translational stop at 1910 AA. TA is a deletion of CCAT's transcription activation domain. (B) Western blot of Neuro2A cells expressing Ca_v_1.2-Gal4 channels depicted in A (upper panel) and Gal4-tagged C-terminal fragments (bottom panel) probed with an antibody to Gal4. (C) Reporter gene activity of Neuro2A cells expressing a UAS-luciferase reporter plasmid along with Ca_v_1.2-Gal4 channels depicted in A or Gal4 alone as a control. Cells were co-transfected with a Renilla luciferase construct driven by the thymidine kinase promoter to control for cell number and transfection efficiency. Results are given as a ratio of Firefly to Renilla luciferase activity. (Means ± SD; *<0.0001 vs. Gal4). (D) Western blot of Neuro2A cells expressing WT, M2011I, and M2078I Ca_v_1.2-Gal4 channels (Upper Panel). Bottom panel shows Gal4-tagged C-terminal fragments. Proteins were detected with a Gal4 antibody. Large molecular protein in channel western represents unsolubilized, multimeric channel proteins. (E) Luciferase activity of Neuro2A cells expressing either Gal4 alone, WT, M2011I, or M2078I Gal4-tagged channels. (Means ± SD; * <0.0001 vs. Gal4). (F) Mean luciferase activity (± SD) in Neuro2A cells expressing CCAT-Gal4 constructs along with the UAS-luciferase reporter and TK-Renilla luciferase construct as controls. CCAT-ΔTA lacks the transcriptional activation domain and serves as a negative control. CCAT-IFIL corresponds to WT sequence. Sequences are included to show the mutations used.

To investigate the mechanisms that generate CCAT in neurons, we first developed a system to monitor its production and transcriptional activity in cells exogenously expressing Ca_v_1.2 (Fig. 1A). We transfected Neuro2A cells with plasmids encoding Ca_v_1.2 fused at its C-terminus with the DNA binding domain of Gal4 (Ca_v_1.2-Gal4), along with the accessory subunits α2Δ and β1b. We used a Gal4 luciferase reporter gene to measure the transcriptional activity of CCAT and Western blot analysis with an anti-Gal4 antibody to measure the production of intact Ca_v_1.2 and its free C-terminal fragment. Western blots of the cell lysates revealed 250 and 40 KD bands, representing full-length Ca_v_1.2 and CCAT, respectively (Fig. 1B). Luciferase measurements of the lysates revealed a 100-fold activation of the luciferase reporter gene relative to cells expressing the Gal4 DNA binding domain alone (Fig. 1C). These results indicate that heterologously expressed Ca_v_1.2 generates CCAT protein and leads to activation of a CCAT reporter gene.

To determine which structural features of the channel are required to generate CCAT, we introduced mutant Ca_v_1.2-Gal4 channels into Neuro2A cells and measured the production of CCAT protein and its transcriptional activity as described above. A deletion of 150 amino acids (AA) of the channel between the end of the last transmembrane domain and the IQ domain prevents the formation of functional channels at the membrane [11]. Surprisingly, this deletion had no effect on the generation of CCAT or on its ability to activate transcription (Fig. 1B and 1C, ΔTM-IQ), suggesting that functional channels are not required for the production or function of CCAT. In agreement with this, we saw no changes in CCAT expression after treating cells with Brefeldin A, a blocker of protein transport that prevents Ca_v_1.2 trafficking to the membrane (Fig. S1A). We next deleted a site on Ca_v_1.2 that is homologous to the putative C-terminal cleavage site in Ca_v_1.1 previously identified by mass spectrometry [10]. Deletion of this site had no effect on the generation of CCAT or on its ability to activate transcription (Fig. 1B and 1C, ΔC.S).

To determine if the production of full length Ca_v_1.2 is necessary for the generation of CCAT, we introduced a stop codon at AA 1910, upstream of the previously characterized CCAT transcriptional activation domain [8]. This mutation prevented the production of the full-length channel but, surprisingly, did not affect the production of CCAT or its ability to activate the luciferase gene (Fig. 1B and 1C, STOP). In contrast, deletion of the final 133 AA of Ca_v_1.2, which includes the transactivation domain of CCAT, completely abolished CCAT expression (Fig. 1B and 1C, ΔTA). These results indicate that the generation of CCAT requires expression of the distal C-terminus of Ca_v_1.2 but does not require production of the full-length channel.

One possible explanation for these results is that CCAT is generated from an alternative translation initiation site at the 3′ end of the Ca_v_1.2 message. Because translation initiation normally begins with methionine, we mutated two methionine residues in the C-terminus of the channel (M2011 or M2078) that could lead to the production of a 20–30 KD C-terminal protein. While mutation of M2078I had no effect on either the generation of CCAT or on its activity as a transcription factor, mutation of M2011 to an isoleucine residue caused an 8 KD decrease in the size of CCAT and completely abrogated CCAT-dependent transcription (Fig. 1D and 1E). These results suggest that CCAT translation begins at M2011.

The loss of transcription in the M2011I mutant suggests that the region between M2011 and M2073 contains a domain that is critical for transcription. A possible candidate is a modified leucine zipper (LZ) composed of the residues IFIL spaced by seven amino acids [12] that is highly conserved from Drosophila to humans (Fig. S1B and S1C). To determine if this domain is necessary for transcription, we mutated the IFIL residues to alanines. Mutation of IL to AA reduced transcription by 75% while mutation of all four residues eliminated transcription completely (Fig. 1F). These results indicate that the LZ in the C-terminus of CCAT is necessary for the transcriptional activity of the protein.

### CCAT is Translated from an Independent Transcript Driven by an Exonic Promoter

**Figure 2:**
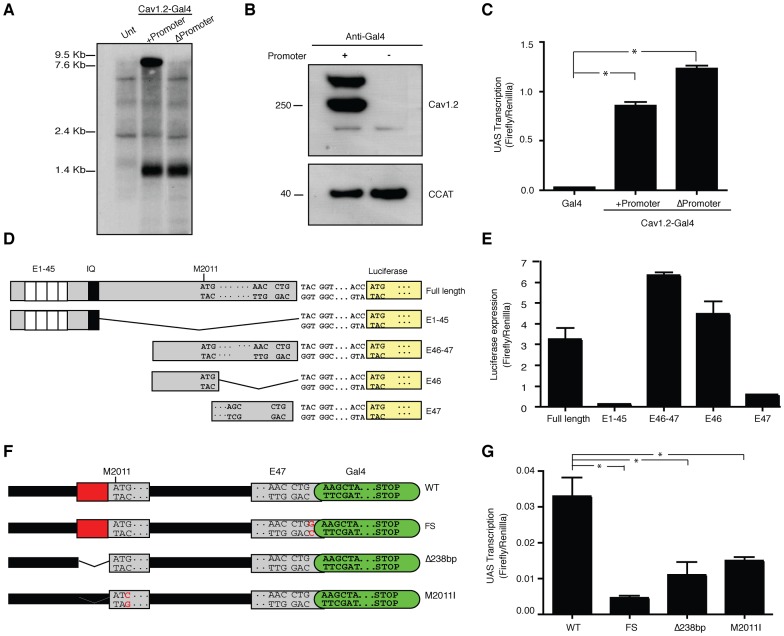
CCAT is Translated from an Independent Transcript Driven by an Exonic Promoter. (A) Northern blot analysis of mRNA extracted from Neuro2A cells expressing Ca_v_1.2-Gal4 channel constructs with or without CMV promoter. The first lane contains mRNA extracted from untransfected cells. The membranes were hybridized with a radioactively labeled RNA probe to Exon 47 of the channel. (B) Western blot of Neuro2A cells expressing Ca_v_1.2-Gal4 channel constructs with or without CMV promoter. Upper panel shows full-length channels. Bottom panels shows Gal4-tagged CCAT. Membranes were immunoblotted with a Gal4 antibody. (C) UAS reporter activity of Neuro2A cells expressing Ca_v_1.2-Gal4 channel constructs with or without CMV promoter. (Means ± SD; *<0.0001 vs. Gal4). (D) Schematic representation of Firefly reporter constructs designed to map the region within the coding sequence of the channel responsible for the promoter activity. Full-length construct has the complete sequence of the channel upstream of the luciferase coding sequence. E1-45 construct includes all exons up to exon 45. E46-47 contains exon 46 and 47 upstream of luciferase. (E) Luciferase activity as a surrogate measure of luciferase expression from constructs depicted in D. Graph compares expression level in Neuro2A cells transfected with either full-length, E1-45, E46-47, E46, or E47 constructs. (Means ± SD). (F) Schematic representation of the minigenes. A 4 Kb genomic segment containing the last two exons and introns of *CACNA1C* was fused to the coding sequence of Gal4. WT is the wild-type minigene. FS is a negative control where a G has been inserted between exon 47 and Gal4. Δ238bp lacks the 238bp region identified in E. Met 2011 has been mutated to isoleucine in M2011. (G) Mean luciferase activity (± SD) in Neuro2A cells expressing WT, FS, Δ238bp, and M2011I Gal4 minigenes along with a UAS-luciferase reporter. (* <0.0001 vs. WT).

CCAT could be translated either from an internal ribosome entry site (IRES) in the Ca_v_1.2 mRNA or from an independent message. To determine if the cells that produce CCAT contain multiple species of mRNAs encoding Ca_v_1.2, we performed Northern blot analysis on lysates from Neuro2A cells expressing Ca_v_1.2-Gal4 channels, using a probe complementary to the last exon of the channel (exon 47). We observed two mRNAs, one of approximately 7.5 Kb corresponding to full-length channel, and a second transcript of approximately 1.4 Kb likely corresponding to CCAT (Fig. 2A). Though this result suggested that a second transcript was involved in generating CCAT, it did not rule out the possibility of an IRES within the full-length channel transcript. To examine this alternative, we deleted the CMV promoter driving the expression of Ca_v_1.2-Gal4 and looked for both the 1.4 Kb message and CCAT protein. As expected, deletion of the CMV promoter led to a loss of the 7.5 Kb channel transcript and the full-length channel protein. However, neither the 1.4 Kb transcript nor the expression of the CCAT protein were affected by deletion of the CMV promoter (Fig. 2A–C, +Promoter and ΔPromoter). Together, these data demonstrate that CCAT is translated from a 3′ mRNA that is generated independently of the mRNA that encodes the full-length channel.

The results above also suggest that CCAT expression is driven by a promoter in the coding sequence of Ca_v_1.2. To identify this promoter, we cloned fragments of the coding sequence of Ca_v_1.2 upstream of the luciferase coding sequence and measured luciferase expression (Fig. 2D and 2E). The full coding sequence of the channel increased luciferase expression approximately 20-fold more than a construct containing exons 1–45 of the channel gene or a reporter gene without a promoter (Fig. 2E, Full length, E1–45). Exons 46 and 47 preserved all the promoter activity of the full length channel, while the 238 base pairs upstream of M2011 in exon 46 preserved 80% of the promoter activity, suggesting that this sequence was sufficient to promote transcription (Fig. 2E, E46-47, E46).

We next examined whether this promoter activity was preserved in the context of the endogenous genomic locus. We constructed a minigene containing 4 Kb of genomic sequence comprising the last two introns and the last two exons of *CACNA1C* and introduced the Gal4 DNA binding domain in frame with the last exon of the gene (Fig. 2F, WT). We introduced this construct into cells along with a UAS reporter gene and found that it activated transcription to eight-fold higher levels than a control plasmid containing a frame shift mutation between CCAT and the Gal4 DNA binding domain (Fig. 2F and 2G, FS). We next deleted the 238 bp promoter region in exon 46 (Δ238bp) and found that this prevented activation of transcription, indicating that this region is also necessary for the production of CCAT in the context of the endogenous gene. Finally, we mutated M2011 in the minigene (Fig. 2F and 2G, M2011I) and found that this prevented activation of the reporter gene, consistent with its role in initiating CCAT translation. A crucial role of M2011 was further confirmed by the observation that mutations of M2073 and 2078, two other methionines in this region, had no effect on the ability of the minigene to activate transcription (Fig. S2A). Taken together, these experiments strongly suggest that exon 46 of Ca_v_1.2 contains a promoter that can drive expression of CCAT, in the context of both the cDNA and the endogenous genomic locus.

### CCAT is Generated From an Independent Transcript *in vivo*

**Figure 3:**
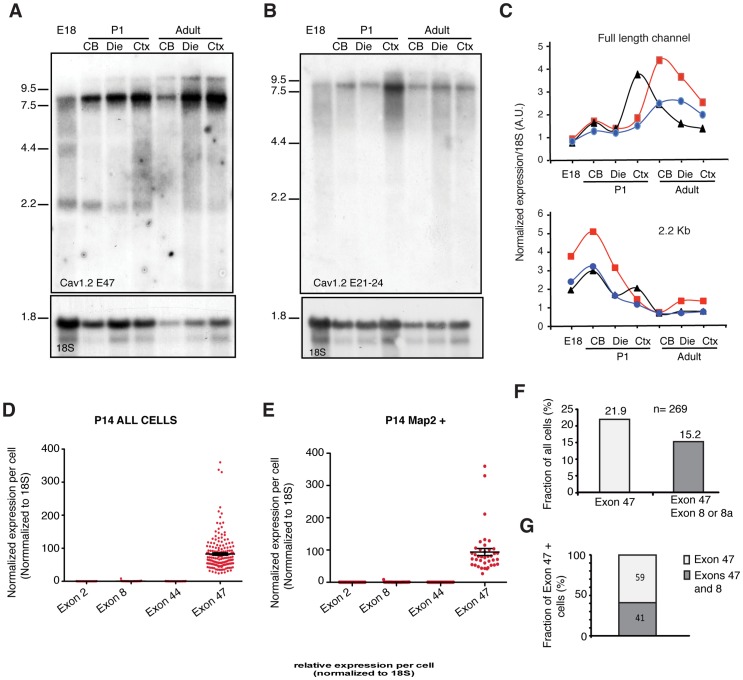
CCAT is Generated From an Independent Transcript *in vivo*. (A–B) Northern blot analysis of mRNA extracted from cortex, diencephalon, and cerebellum from E18, P1, and adult rats. The membranes were hybridized with radioactively labeled DNA probe to either exon 47 (A) or exons 21–24 (B) of the channel. Bottom panel shows the same membrane labeled with a probe to the 18S ribosomal RNA as a loading control. (C) Graphs showing the normalized expression of the full-length and 2.2 Kb band in the cortex, midbrain, and cerebellum at 3 developmental stages: E18, P1, and adult. Each line represents an independent experiment. The northern blot shown in A corresponds to the blue tracing. Signals were normalized to the 18S RNA signal. (D–E) Dot plots showing the normalized expression of exons 2, 8, 44, and 47 per cell in mouse neurons. For all comparisons of exon 47 to the other exons, p<0.001 (***) according to one-way ANOVA with post-hoc Bonferroni correction for multiple comparisons. Exons 2, 8, and 44 are not significantly different from each other. (F) Graphs showing fractions of human IPSC-derived neurons expressing exon 47 exclusively or both exons 47 and 8 (n = 269). (G) Fraction of exon 47-containing human IPSC-derived neurons expressing either exon 47 exclusively or co-expressing exon 8.

We next investigated whether multiple mRNAs were also generated from the *CACNA1C* gene *in vivo*. We extracted mRNA from the cerebral cortex, diencephalon, and cerebellum of rats at different developmental stages and analyzed them using Northern blotting. We used a probe targeting exon 47 to detect both the CCAT and Ca_v_1.2 mRNAs and found two distinct transcripts, one of 8.5 Kb that likely encodes the full length channel and a second one of 2.2 Kb that could encode for CCAT (Fig. 3A). A second probe directed against exons 21–24 detected only the full-length channel (Fig. 3B). Interestingly, in three independent experiments, the CCAT and Ca_v_1.2 transcripts were regulated reciprocally during development and in different brain regions. CCAT expression was highest in E18 brains and P1 cerebellum, whereas the full-length channel reached peak expression in adult diencephalon and cortex (Fig. 3C). These data confirm the existence of at least two different exon 47-containing mRNAs *in vivo,* and is consistent with the previously reported idea that CCAT may negatively regulate transcription of Ca_v_1.2 channels [9].

We next asked whether an mRNA containing exon 47 is expressed independently of the full Ca_V_1.2 mRNA in individual neurons using single cell quantitative PCR. We isolated single cells from P14 forebrain and measured the abundance of messages containing exons 2, 8, 44, and 47 using Fluidigm dynamic arrays. In all cells, messages containing exon 47 were approximately 80-fold more abundant at the single cell level than messages containing the other exons (n = 182; Fig. 3D). Analysis of the cells co-expressing the neuronal marker Map2 showed 100-fold higher expression of exon 47 (n = 39; Fig. 3E). These data suggest that this exon is expressed independently of the full channel mRNA. Additional support for this conclusion came from single cell quantitative PCR analysis of induced-pluripotent-stem-cell (iPSC)-derived human neurons (Fig. S3A). We found that 21.9% of cells expressed exon 47 without expressing other exons, while 15.2% expressed both exon 47 and other Ca_v_1.2 exons (Fig. 3F and 3G,). Interestingly, the oligodendrocyte marker CMTM5 and the inhibitory neuronal markers GAD67 and NKX2.1 were significantly correlated in cells expressing exon 47 alone, suggesting that CCAT or another exon 47-containing message may be important for the function or specification of these cells (Fig. S3B). Together, these data provide further support for the existence of 3′ *CACNA1C* transcripts and indicate that these transcripts are regulated in a cell-type specific manner.

### CCAT Expression is Regulated During Brain Development

**Figure 4:**
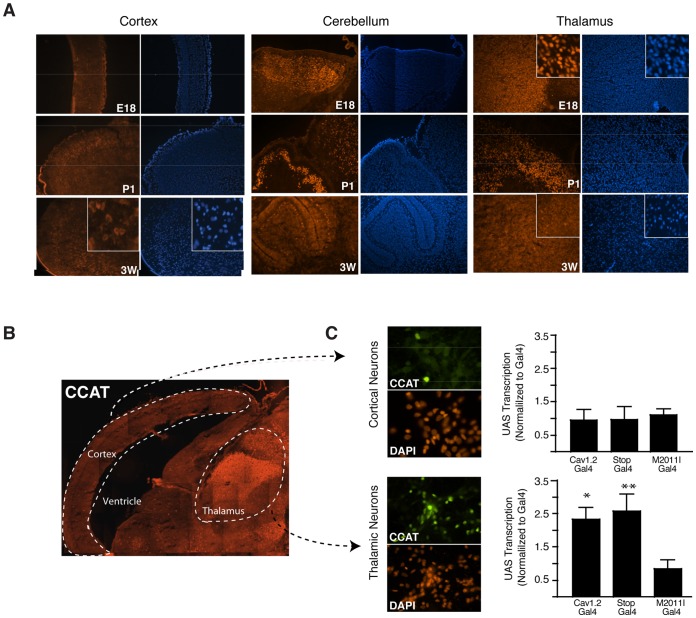
CCAT Expression is Regulated During Brain Development. (A) Immunohistochemistry of E18, P1, and 3-week-old rat cortex, cerebellum, and thalamus showing developmental variation in the amount and distribution of CCAT nuclear staining. Cortex shows mostly cell body and dendritic staining of CCAT in the three developmental stages. Anti-CCAT is shown in red and nuclei in blue. (B) Immunohistochemistry of E18 mouse sagittal sections through the cortex, ventricle, and thalamus stained with anti-CCAT antibody. (C) Immunocytochemistry of cortical and thalamic neurons grown 5 days *in vitro* stained with anti-CCAT. Transcriptional assays of cortical (top) and thalamic (bottom) neuronal cultures transfected with the UAS-luciferase reporter along with the Gal4-tagged channels described in Fig. 1. Bars represent normalized transcription to Gal4 alone. (Means ± SD; * <0.005 and ** <0.0001 vs. M2011I-Gal4).

To provide additional evidence for the idea that CCAT is differentially regulated during development and in different cell types, we used anti-CCAT antibodies to probe sections of cerebellum, thalamus, and cortex from developing rats. The fraction of cells with CCAT nuclear staining decreased with age in the cerebellum and thalamus. In E18 embryos, most cells in the thalamus and developing cerebellum had nuclear CCAT, whereas in three-week old rats, the staining in the cerebellum was confined to a thin layer of Purkinje neurons and modest nuclear staining in the thalamus (Fig. 4A and 4B). We detected almost no nuclear CCAT staining in the cortex at any stage of development. Together, these results show that the distribution and abundance of nuclear CCAT is consistent with the distribution of the 2.2 Kb transcript.

The tissue-specific expression of CCAT implies that the CCAT promoter is differentially regulated in different cells. To test this idea, we compared the activation of the CCAT promoter using Ca_v_1.2-Gal4 channels and the UAS-luciferase reporter gene in primary cultures of E18 thalamic and cortical neurons (Fig. 4C). Both the full length Ca_v_1.2-Gal4 and Ca_v_1.2-Stop-Gal4 activated transcription in thalamic neurons but not in cortical neurons, consistent with the idea that the CCAT promoter is more active in the developing thalamus than in the developing cortical plate. As expected, the Ca_v_1.2-Gal4 M2011I mutant channel did not activate transcription in thalamic cells, indicating that transcription depends on translation initiation at this methionine. These results indicate that CCAT protein expression is well correlated with the activity of the CCAT promoter in cells.

### Transcriptional Start Sites in the 3′ End of *CACNA1C* Produce Multiple Proteins

**Figure 5:**
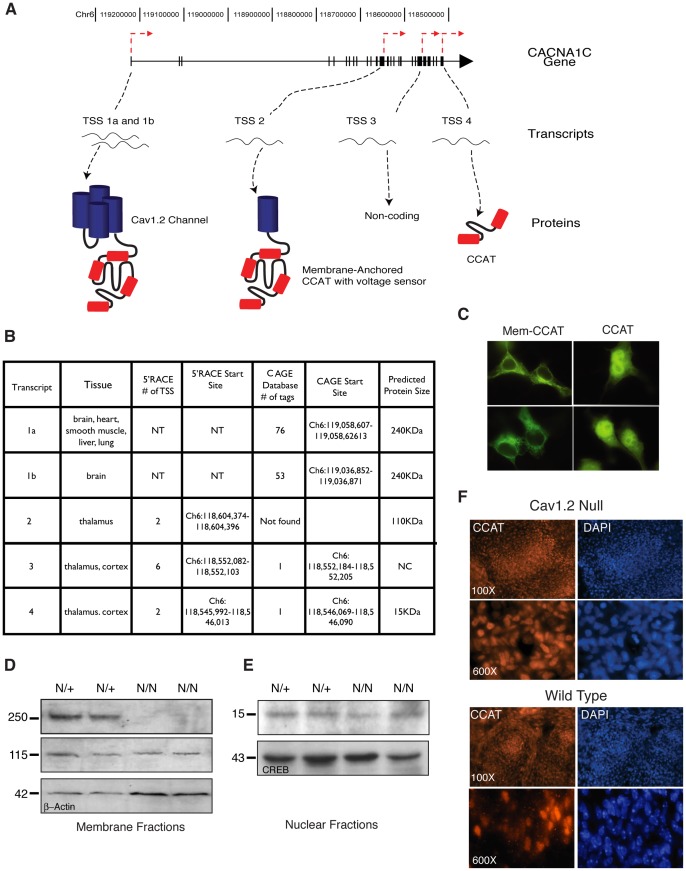
Transcriptional Start Sites in the 3′ End of *CACNA1C* Produce Multiple Proteins. (A) Schematic representation of *CACNA1C* showing the location of TSS's found and depictions of the proteins predicted to be expressed from these transcripts. (B) Table showing a summary of transcriptional start sites and nearby CAGE tags. Chromosomal addresses for experimental TSS are given as the corresponding location in the Mouse July 2007 genome assembly. NT (Not tested), NC (non-coding). (C) HEK cells expressing Mem-CCAT GFP and CCAT GFP constructs. (D-E) Western blot analysis of membrane fractions (D) and nuclear fractions (E) obtained from 11.5 dpc heterozygous (N/+) or homozygous (N/N) Ca_v_1.2 knockout embryos probed with the anti-CCAT antibody. Bottom panels show loading controls. (F) Immunohistochemistry of 11.5 dpc Ca_v_1.2 null embryos reveals strong nuclear staining with the anti-CCAT antibody (red) in the developing somites. Nuclei are shown in blue.

The results above indicate that there are multiple mRNA transcripts derived from the *CACNA1C* gene. To map the transcriptional start sites (TSS) within the gene, we sequenced the 5′ ends of mRNAs found from E18 rat brain using Rapid Amplification of cDNA Ends (5′ RACE). To test our approach, we first determined the TSS in the transcript generated in cells expressing the Ca_v_1.2-Gal4 and Ca_v_1.2-Gal4 promoter-less constructs. The TSS was 50 bp upstream of M2011 (Fig. S4A, 5A, and 5B). We then tested mRNA isolated from E18 rat brain. In five independent 5′-RACE experiments, we identified three novel TSS's in the 3′ region of the *CACNA1C* gene that are independent of the two known start sites of the channel (TSS 1 and 1b). TSS 2 gives rise to a transcript that is predicted to encode a protein with a molecular weight of 110 KD that contains the channel's sixth transmembrane helix of ion pore domain III, domain IV, and the C-terminus. Introduction of this mRNA fused to GFP into mammalian cells led to the production of a 120 KD membrane-bound protein, indicating that this mRNA can be translated in cells (Fig. 5C). TSS 3 is predicted to produce a noncoding RNA, and TSS 4 encodes for a 15 KD protein predicted to be full-length CCAT. Expression of this final mRNA fused to GFP in cells leads to the production of a soluble protein that is localized to the nucleus and looks in all respects like CCAT (Fig. 5C). TSSs 3 and 4 can also be found in the CAGE database, a genome wide database of mouse transcriptional start sites, providing additional support for the existence of these sites in multiple species. These results strongly suggest that the *CACNA1C* gene has multiple start sites that lead to the production of proteins with dramatically different structural and functional features.

One prediction of our findings is that mice lacking 5′ exons of *CACNA1C* should not express full-length Ca_v_1.2 channels but should express other proteins derived from 3′ regions of the gene. Our 5′ RACE experiments suggest the existence of two additional protein products a 110 KD protein from TSS 2 and CCAT, a 15 KD protein from TSS 4. To test this idea, we used Western blotting and immunocytochemistry to measure nuclear CCAT expression in the brains of heterozygous (N/+) or null (N/N) embryos lacking exons 14 and 15 of the *CACNA1C* gene at 11.5 days post-conception (dpc) (Fig. S4C and S4D). Antibodies directed against the C-terminus of Ca_v_1.2 revealed a 250 KD and a 115 KD band in the membrane fraction of the brain lysates of WT mice and a 15 KD band in the nuclear fractions (Fig. 5D and E). These three bands have the same molecular weights as the three CACNA1C-derived proteins that arise from TSSs 1, 2, and 4, suggesting that they might be products of this gene. Mice lacking exons 14 and 15 of *CACNA1C* are missing the 250 KD band but still generate the 115 and 15 KD proteins. This result is consistent with the idea that the loss of 5′ exons disrupts production of the full-length channel but does not alter the production of proteins encoded by 3′ exons in the gene. To provide further evidence for the persistence of CCAT in Ca_v_1.2 knock out mice, we performed immunocytochemistry in N/+ and N/N embryos using anti-CCAT antibodies. N/+ embryos had cytoplasmic and membranous anti-CCAT immunoreactivity in the developing neural tissue, heart muscle, and major blood vessels. Strong nuclear staining was noted in somites and mesenchymal tissue (Fig. S4E). Ca_v_1.2 null embryos showed intact nuclear staining in somites (Fig. 5F), suggesting that anti-CCAT reactive nuclear protein was still present in cells lacking full-length channel.

## Discussion

In this study, we provide evidence supporting that CCAT is produced not by cleavage of the Ca_v_1.2 channel protein but by an internal promoter in the *CACNA1C* gene that drives expression of exons 46 and 47 independently. This is unusual in at least two respects. First, although alternative promoters have been described for many genes, they generally regulate the production of similar proteins. In this case, the promoter controls the production of a 15 KD transcription factor from a gene that also produces a 240 KD calcium channel. This kind of genetic multitasking, while common in viruses, is rare in mammals. Second, the promoter for CCAT is located in the coding region of the *CACNA1C* gene. An exonic promoter has been described for the sperm-specific axonemal dynein subunit Sdic in Drosophila, but in that case the promoter is contained in the coding region of the neighboring gene AnnX, [13]. While this is the first report of this kind of promoter in mammals, whole genome transcriptome and transcription factor binding studies suggest that exonic promoters could be more widespread in the genome than we have appreciated.

The unusual relationship between CCAT and Ca_v_1.2 suggests a novel mechanism for the regulation of channel expression. Our studies show that CCAT and Ca_v_1.2 are expressed in complementary patterns in the developing brain. CCAT is highly expressed in the developing thalamus and cerebellum and is suppressed later in development, while Ca_v_1.2 is expressed in a reciprocal pattern. Consistent with this, we found exon 47 to be exclusively expressed in a significant number of human iPSC-derived neurons in the absence of other Ca_v_1.2 exons. This is consistent with the recent finding that CCAT suppresses the expression of Ca_v_1.2 and suggests that CCAT may play a role in controlling channel expression during development.

Our findings that internal promoters reside within the *CACNA1C* gene and that multiple proteins can be expressed from the same gene have potential implications for neuropsychiatric diseases. Recent genome-wide association studies have identified *CACNA1C* as a candidate gene in bipolar disorder, schizophrenia, and autism. It has been assumed that mutations in this gene affect the function or expression of the channel, but it is possible that they could also alter the levels or function of CCAT or the other proteins generated from this gene.

In summary, these results provide insights into a mechanism through which complexity is generated in mammalian genomes. Exonic promoters that lead to the expression of protein fragments increase the diversity of the proteome, which has implications for the prediction of gene products and for the interpretation of knockout phenotypes.

## Materials and Methods

### Materials

For details on reagents, antibody generation, and dilutions see Supporting Information.

### Cell Culture and Transfection

Neuro2A cells were cultured in Dulbecco's Minimal Essential Media (DMEM) containing 10% fetal bovine serum (FBS; 15% for PC12s), penicillin, streptomycin (P/S), and L-glutamine (LQ).

Cortical and thalamic neurons were dissociated from E17–19 Sprague Dawley rats as described (Xia et al., 1996) and maintained for 6–14 days in culture in Basal Medium Eagle with 5% FBS, P/S, LQ, and 1% glucose. For a detailed description see Supporting Information.

### Plasmid Construction

For details on plasmid construction including primers see Supporting Information.

### Subcellular Fractionation

#### Note

For biochemical experiments all protein samples were kept at or below −4°C. Complete protease inhibitor tablets were added to all solutions fresh before the experiment.

For Ca_v_1.2 knockout mice and the controls, pregnant females at 11 days post plugging were anesthetized using CO_2_ and embryos were dissected in cold PBS. The tails were cut and separated for DNA genotyping. Tissue subcellular fractionation and protein extraction was performed as described [14]. Briefly, after dissection, embryos were cut and washed in cold 250-STMPBS, homogenized, and centrifuged at 800 g for 15 min. The nuclear pellet was resuspended and homogenized again. Supernatants were pooled to form cytosolic fraction I and the pellet constituted nuclear fraction I. The cytosolic fraction was centrifuged 1 h at 100,000 g to pellet the microsomal fraction, which was solubilized in ME buffer for 1 h and finally centrifuged for 30 min at 9000 g. This supernatant constituted the solubilized membrane fraction. Nuclear fraction I was resuspended in 2M-STMDPS buffer and layered on a 2M STM cushion in a centrifuge tube. Samples were spun at 80,000 g for 30–45 min. The pelleted nuclei were solubilized in NE buffer, then passaged through an 18 gauge needle and spun at 9000 g for 30 min. Supernatant was used as salt soluble nuclear proteins.

### Immunoprecipitation and Western Blotting

Neuro2A cells were lysed 24 hrs post-transfection using lysis buffer containing 1.5% TritonX-100, 50 mM TRIS-HCL pH 7.5, 150 mM NaCl, 10mM EDTA, and protease inhibitor tablets (Roche). Immunoprecipitations were carried out using 4 µg of Gal4 antibody and Protein A/G beads (Santa Cruz).

Western blotting was conducted using standard protocols. Antibodies and dilutions are included in Supporting Information. Protein concentration was measured by the BCA method (Pierce).

### Immunofluorescence

Cortical and thalamic neurons were fixed in 4% paraformaldehyde/2% sucrose and 10 mM EDTA in PBS for 10 minutes followed by permeabilization with 0.025% TritonX-100 and blocking with 3% bovine serum albumin (BSA) in phosphate-buffered saline (PBS). For details on antibody dilutions see Supporting Information.

Brains from embryonic and postnatal rats and wildtype C57BL/6 mice were dissected and fixed in 4% paraformaldehyde (PFA) in 0.1M phosphate buffer (PB, pH 7.4). Brains from animals younger than embryonic day 18 (E18) were fixed in PFA for 30 minutes, whereas brains from postnatal mice were fixed overnight at 4°C. Subsequently, brains were transferred to a 30% sucrose solution overnight for cryoprotection, embedded in Tissue-Tek OCT compound, and cut (15 μm for embryonic brains, 25–30 μm for postnatal brains) on a freezing cryostat (Leica, CM3050). All tissue was stored at −80°C until further use. For immunofluorescence analysis of CCAT expression, slides were washed in PBS containing 0.1% bovine serum albumin (BSA) to remove excess OCT. Sections were blocked in 10% normal goat serum (NGS; Gibco) containing 0.25–3% Triton X-100 for 1 hour at 25°C. Primary antibody (anti-CCAT, 1∶150) was applied overnight in 10% NGS with 0.1% Triton X-100 at 4°C followed by the appropriate fluorochrome conjugated secondary antibody (Alexa conjugates; Molecular Probes) for 1 hour at 25°C. Slides were then washed in PBS with 0.1% BSA, counterstained with Hoechst or DAPI, and mounted in Aqua Poly/Mount (Polysciences, Inc.) for fluorescent microscopy.

Slides were visualized by conventional epifluorescence microscopy using a cooled CCD camera (Hamamatsu) coupled to an inverted Nikon Eclipse E2000-U microscope.

### Luciferase Assays

For transfection ratios see Supporting Information. For all luciferase assays the mean of three biological replicates is reported. Most luciferase assays were performed 24 hr after transfection using the Dual-Glo luciferase assay kit from Promega. A Veritas 96-well luminometer (Turner Biosystems) was used to measure light emission. PFA-CMV (Gal4 alone) and UAS-luciferase constructs were obtained as part of the PathDetect transreporting system (Strategene). Data sets were analyzed using Igor Pro and Prism4 software. Two-paired t tests were performed between relevant conditions.

### Northern Blots

mRNA was extracted from rat brains and N2A cells using the Fast-Track mRNA isolation kit according to manufacturer's instructions (Invitrogen). Northern blots were carried out using the NorthernMax kit solutions and followed the protocol as recommended by the manufacturer (Ambion), using 5 µg of mRNA. For more details including probe synthesis see Supporting Information.

### Single Neuron Isolation from Mouse Brain

Mouse forebrain neurons were manually dissected, dissociated using papain, and stained with propidium iodine. Clone sorting in 96-well PCR plates (Eppendorf) was performed at the Stanford Shared FACS Facility on a BD Influx cell sorter.

### iPSC Generation and Maintenance

iPSC lines were generated as previously described [15]. Briefly, cells were derived from two healthy controls using infection with retroviruses expressing SOX2, OCT3/4, KLF4, and C-MYC of dermal fibroblasts. The iPSC lines were extensively characterized using a set of assays, including immunostaining with anti-Nanog and Alkaline Phosphatase antibodies, karyotyping, generation of teratomas in nude mice, mapping of viral integration sites, and Illumina arrays. iPSC were cultured on irradiated DR4 mouse embryonic fibroblast feeders in the following iPSC media: DMEM/F12 (1∶1) media (Invitrogen) containing 20% knockoutTM SR (Invitrogen), 1 mM non-essential amino acids (Invitrogen), 3 mM L-glutamine (Invitrogen), 0.1 mM β-mercaptoethanol (Sigma-Aldrich), 100 units/ml penicillin and 100 μg/ml streptomycin (Invitrogen), and 10-15 ng/ml bFGF (R&D Systems).

### Neuronal Differentiation

Neuronal differentiation was carried out using a modified version of a previously described protocol [16]. To generate embryoid bodies (EBs), iPSC colonies were detached by incubation with dispase (Invitrogen). EBs were kept in suspension in iPSC media in low attachment plates (Corning) for 4 days. For neural induction, day 5 EBs were plated in Neural Media (NM) on polyornithine/laminin coated plates with FGF2 (20 ng/µl) and EGF (20 ng/µl). NM media contained: Neurobasal (Invitrogen, cat. no. 10888), B-27® Supplement Minus Vitamin A (50X, cat. no. 12587-010), GlutaMAX (Invitrogen, 100x), 100 units/ml penicillin, and 100 μg/ml streptomycin (Invitrogen). Attachment of the EBs was facilitated by a 12 hour pulse with 10% FBS. During neural induction, the NM media supplemented with growth factors was changed every day. Rosettes were picked mechanically and immediately transferred to low attachment wells (Corning) in NM with FGF2 (20 ng/µl) and EGF (20 ng/µl), and kept in suspension for 7 days. Media changes on the neurospheres were performed every other day, and mechanical trituration was performed on days 3 and 5. For neural differentiation, ∼10 neurospheres were plated on 12- or 15 mm polyornithine/laminin coated coverslips in NM media supplemented with FGF2 (20 ng/µl) and EGF (20 ng/µl) for the first 24 hours, and the media was then changed to NM supplemented with BDNF (20 ng/µl) and NT3 (20 ng/µl). Half of the media was changed every other day for 21–22 days. Single cell qPCR arrays from the neuronal cultures were performed at day 43 of differentiation.

### Single Cell qPCR

The Fluidigm Dynamic Array [17] was used to measure the expression of 96 genes in single cells. Neuronal cultures at day 43 of differentiation were rinsed with HBSS and incubated with Accutase (StemCell Technologies) until they detached from the dish. After one wash with fresh Neurobasal medium (Invitrogen), cells were resuspended in Neurobasal/B27 medium containing 1 μg/ml propidium-iodide (Molecular Probes) and filtered through a 40 μm nylon cell strainer (BD Biosciences). Cells were sorted into 10 μl pre-amplification mix containing 40 nM of all primers for the 96 genes of interest, and the following components of the CellsDirect One-Step qRT-PCR kit (Invitrogen): 2x reaction mix, SuperScript III RT/Platinum Taq mix. After sorting, samples were reverse transcribed and pre-amplified for 18 cycles. Pre-amplified samples were diluted (2x) with TE buffer and stored at −20°C. Sample and assay (primer pairs) preparation for 96.96 Fluidigm Dynamic arrays was done according to the manufacturer's recommendation. Briefly, sample was mixed with 20x DNA binding dye sample loading reagent (Fluidigm Corp.), 20x EvaGreen (Biotium), and TaqMan® gene expression master mix (Applied Biosystems). Assays were mixed with 2x assay loading reagent (Fluidigm Corp.) and TE to a final concentration of 5 μM. The 96.96 Fluidigm Dynamic Arrays (Fluidigm Corp.) were primed and loaded on an IFC Controller HX (Fluidigm Corp.) and qPCR experiments were run on a Biomark System for Genetic Analysis (Fluidigm Corp.). Data was collected and analyzed using Fluidigm Real-Time PCR Analysis software (v2.1.3 and v3.0.2). Further data analysis was performed using Microsoft Excel. Every experiment contained samples for 5 standard dilutions of a mixed human cDNA library, a negative control, and cDNA from 90 single cells. Cells were identified based on RSG18 expression, encoding the 18 S small ribosomal subunit. In total, three 96.96 chip experiments were performed using neurons differentiated from three iPSC lines. Individual cell expression values were normalized by 18 s for all primer pairs. Fold differences were also normalized by efficiency of each primer pair.

For a list of primers used see Supporting Information.

### 5′ RACE Experiments

5′ RACE was carried out using Invitrogen's GeneRacer kit according to manufacturer's instructions. For experimental details see Supporting Information.

### Generation of Conditional Knockout Mice for the Ca_v_1.2 Calcium Channel Gene

Mice lacking the Ca_v_1.2 calcium channel gene were generated by homologous recombination mediated gene targeting (Fig. S1B and 1C). The targeting construct was designed to delete exons 14 and 15 under the control of Cre recombinase. To facilitate Southern screening, a new BamHI site was generated 5′ to Exon 16, and the original BamHI site 5′ to Exon 14 was eliminated in the targeting construct. The Ca_v_1.2 targeted mice were maintained in a 129/sv-C57BL/6 mixed background. The Ca_v_1.2 floxed (Neo deleted) mice were generated by crossing Ca_v_1.2 targeted mice with FLpe transgenic mice (kindly provided by Dr. Dymecki). Ubiquitous deletion of the floxed exon was achieved using CMV-CRE*-*transgenic mice.

### Ethics Statement

All mouse experiments were conducted as described in our mouse protocol, which was approved by the Stanford University Administrative Panel on Laboratory Animal Care (APLAC, protocol #13705). iPSCs were generated using approved procedures under SCRO protocol #232.

## Supporting Information

Figure S1 **(A) Mean luciferase expression in Neuro2A cells transfected with the UAS-luciferase reporter and either Gal4 alone or Ca_v_1.2-Gal4 channels.** Channel-expressing cells were treated with 10 μM Brefeldin A for 3–6 h. (B) Sequence alignment of the c-termini of the L-type calcium channel family. Sequences for Ca_v_1 channels from zebrafish, mouse and human were aligned to the ancestral *C. elegans* and *D. melanogaster* L-type channels. Colors represent similarity based on percentage identity. Two regions of conservation are identified: the sequence surrounding the IQ domain and the modified leucine zipper domain. (C) Sequence alignment focused on mouse Ca_v_1 channels and C. *elegans* and *D. melanogaster* L-type channels.
(PDF)

Figure S2 **Mean luciferase activity (± SD) in Neuro2A cells expressing minigenes in which either all or two of the three possible methionines were mutated.**
(PDF)

Figure S3 **(A) Pie chart showing fractions of 269 human IPSC-derived neurons expressing: Ca_v_1.2 channel exons, exon 47 alone, no exon 47 and exon 47 co-expressed with exon 8.** (B) Correlations of exon 47 expression with other neuronal markers in human IPSC-derived neurons. Gsx/Gsh2 is involved in ventral telencephalon fate specification. CMTM5 is an oligodendrocyte marker. FoxP1 marks deeper layer cortical neurons and striatal projection neurons. GAD67 marks inhibitory interneurons. NKX2.1 is a global marker of ventral forebrain identity and cortical interneuron progenitors. DLX1 marks ventral inhibitory neurons. MSX2 marks neural crest derivatives and is also expressed in the midbrain.
(PDF)

Figure S4 **(A) Schematic representation of the 5′RACE approach to determine the TSS for the CCAT transcript generated from Ca_v_1.2-Gal4. Briefly, two sequential phosphatase treatments are used to inactivate truncated or non-mRNAs and prepare intact, originally capped mRNAs for ligation of an RNA oligo to the uncapped 5′ end.** In this experiment, reverse transcription was performed with a Gal4 reverse primer. Nested primers within the 5′ tag and the Gal4 coding sequence were used for PCR. The bands were then cloned and sequenced. (B) Agarose gel of PCR products amplified after performing 5′ RACE, as described in A, of Neuro2A cells expressing Ca_v_1.2-Gal4 channels with and without the CMV promoter. (C) Schematic of the Ca_v_1.2 knockout strategy. (D) Southern blot showing the efficacy of recombination and the expected molecular weight of the BamHI-digested genomic fragments after recombination. (E) Immunohistochemistry of heterozygote 11.5 dpc embryos stained with anti-CCAT antibody. Membranous staining is seen in the developing cortex and heart muscle wall (HM). Staining is noticeably nuclear in somites, mesenchymal cells and blood vessels. CCAT staining is not detected in the liver.
(PDF)

Methods S1 **Description of reagents and antibodies used, plasmid generation, cell culture and transfection, luciferase assay transfection, Northern blots, single cell qPCR primers, 5**′ **RACE experiments, sequence analysis and multiple sequence alignments.**
(DOC)
