# Supplementary material for: A Promoter in the Coding Region of the Calcium Channel Gene CACNA1C Generates the Transcription Factor CCAT
Source: PLoS One. 2013 Apr 16;8(4):e60526. doi: 10.1371/journal.pone.0060526 (PMC3628902; doi:10.1371/journal.pone.0060526)
Supplement: Figure S1 — (A) Mean luciferase expression in Neuro2A cells transfected with the UAS-luciferase reporter and either Gal4 alone or Cav1.2-Gal4 channels. Channel-expressing cells were treated with 10 μM Brefeldin A for 3–6 h. (B) Sequence alignment of the c-termini of the L-type calcium channel family. Sequences for Cav1 channels from zebrafish, mouse and human were aligned to the ancestral C. elegans and D. melanogaster L-type channels. Colors represent similarity based on percentage identity. Two regions of conservation are identified: the sequence surrounding the IQ domain and the modified leucine zipper domain. (C) Sequence alignment focused on mouse Cav1 channels and C. elegans and D. melanogaster L-type channels. (PDF) [file pone.0060526.s001.pdf]

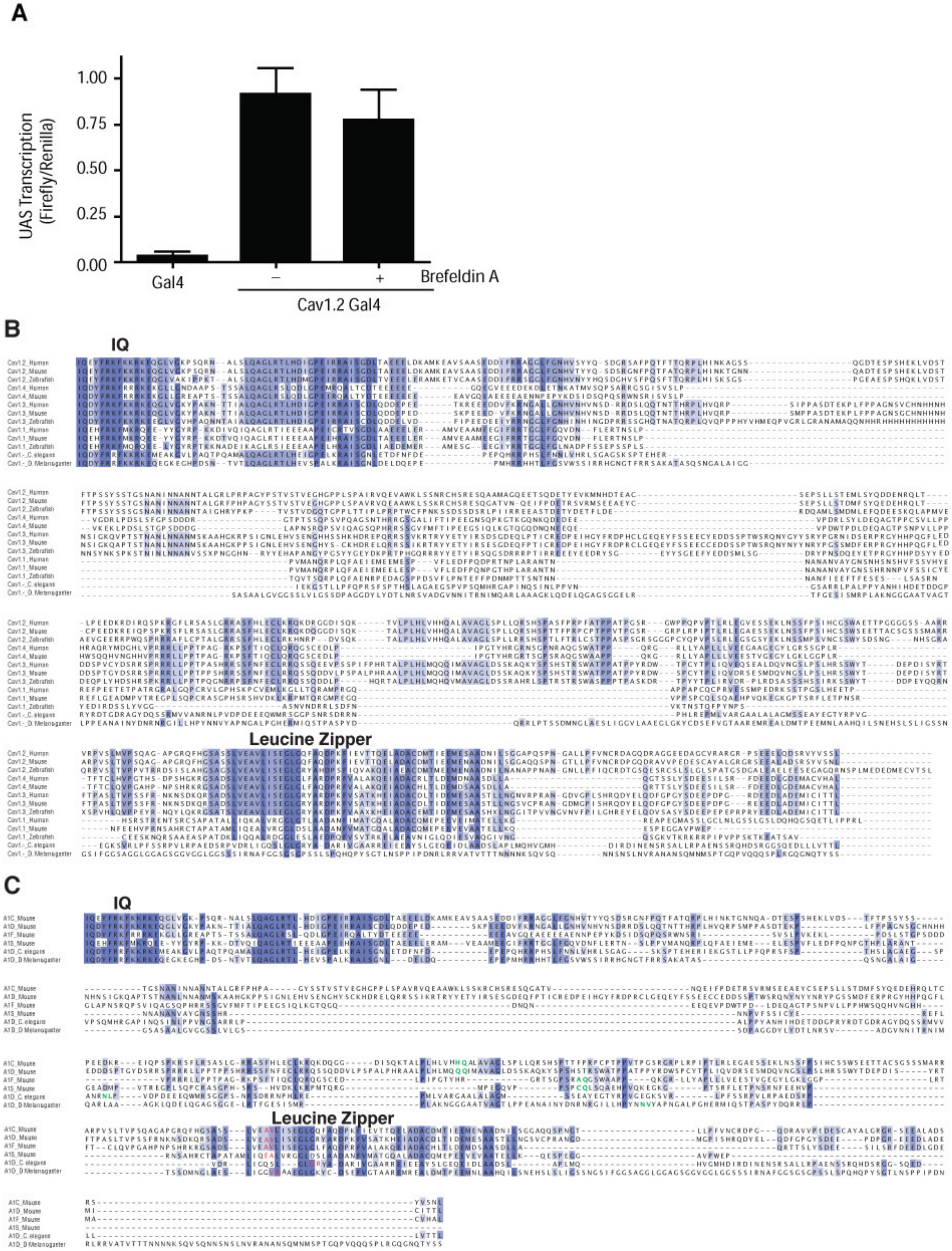
A
UAS Transcription (Firefly/Renilla)
1.00
0.75
0.50
0.25
0.00
Gal4
−
+
Brefeldin A
Cav1.2 Gal4
B
IQ
Leucine Zipper
C
IQ
Leucine Zipper
A1C_Mouse
A1D_Mouse
A1F_Mouse
A1S_Mouse
A1D_C.elegans
A1D_D.Melanogaster
