# Supplementary figures and images for: A Promoter in the Coding Region of the Calcium Channel Gene CACNA1C Generates the Transcription Factor CCAT
Source: PLoS One. 2013 Apr 16;8(4):e60526. doi: 10.1371/journal.pone.0060526 (PMC3628902; doi:10.1371/journal.pone.0060526)

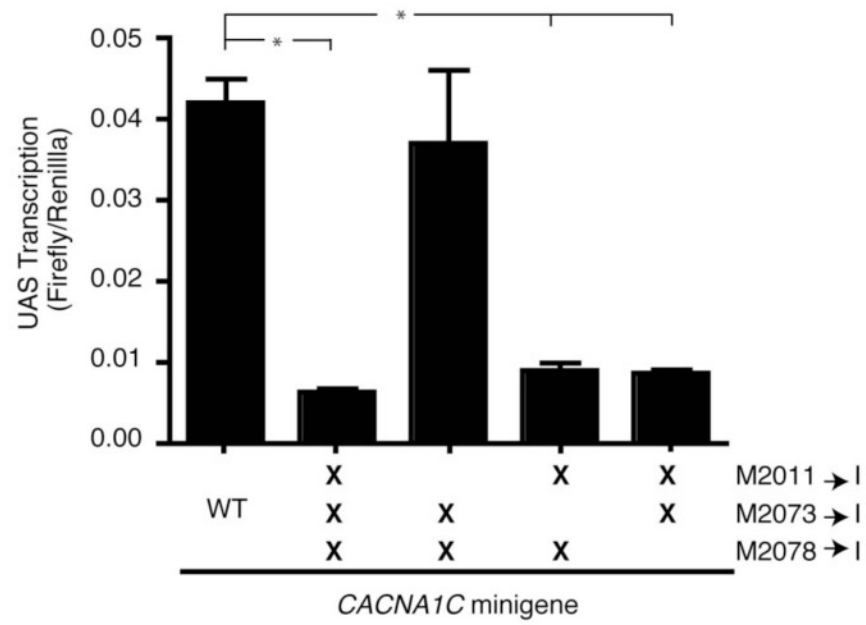

Supplement: Figure S2 — Mean luciferase activity (± SD) in Neuro2A cells expressing minigenes in which either all or two of the three possible methionines were mutated. (PDF) [file pone.0060526.s002.pdf]

A

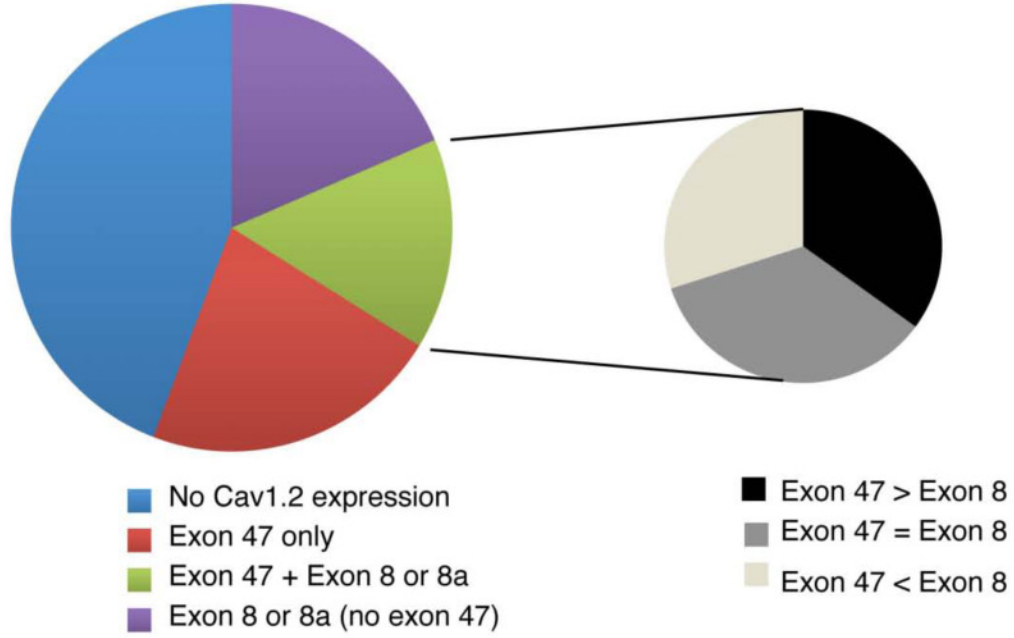

B

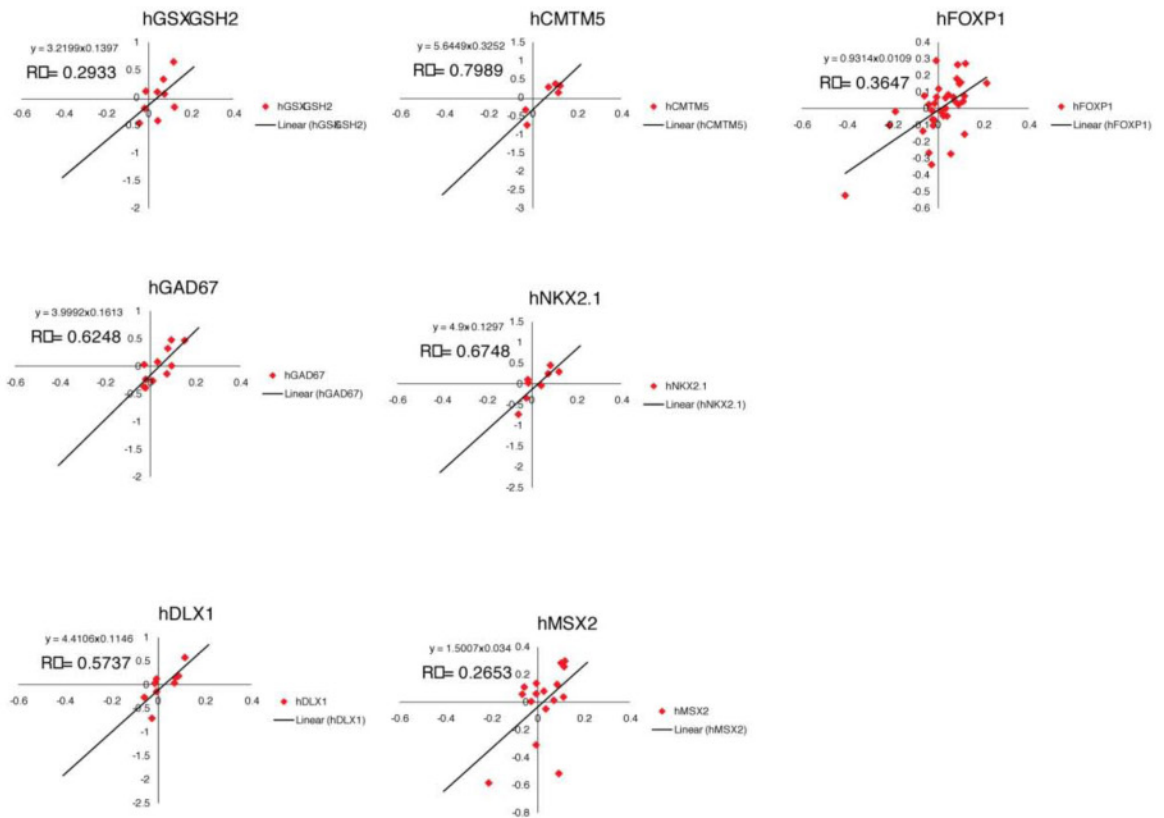

Supplement: Figure S3 — (A) Pie chart showing fractions of 269 human IPSC-derived neurons expressing: Cav1.2 channel exons, exon 47 alone, no exon 47 and exon 47 co-expressed with exon 8. (B) Correlations of exon 47 expression with other neuronal markers in human IPSC-derived neurons. Gsx/Gsh2 is involved in ventral telencephalon fate specification. CMTM5 is an oligodendrocyte marker. FoxP1 marks deeper layer cortical neurons and striatal projection neurons. GAD67 marks inhibitory interneurons. NKX2.1 is a global marker of ventral forebrain identity and cortical interneuron progenitors. DLX1 marks ventral inhibitory neurons. MSX2 marks neural crest derivatives and is also expressed in the midbrain. (PDF) [file pone.0060526.s003.pdf]

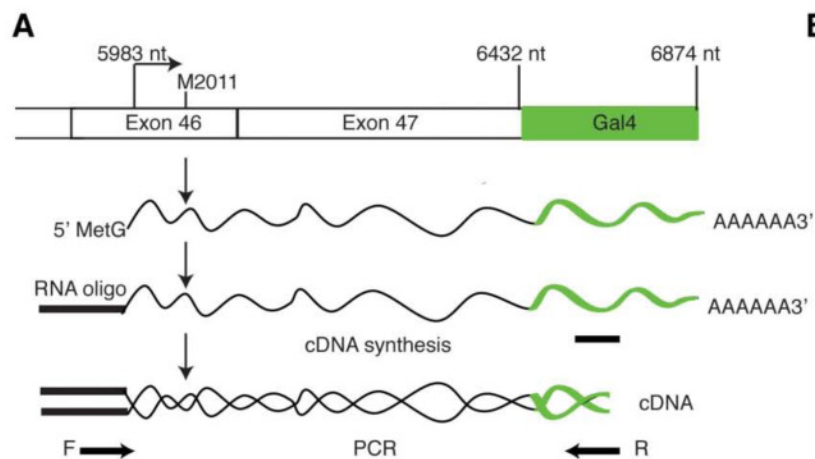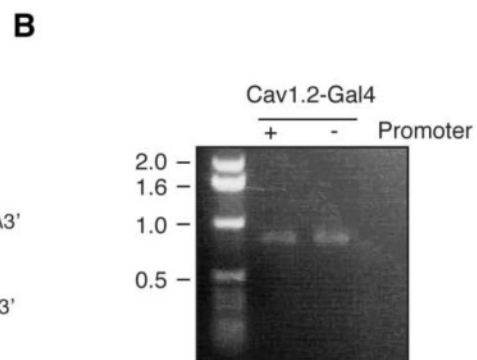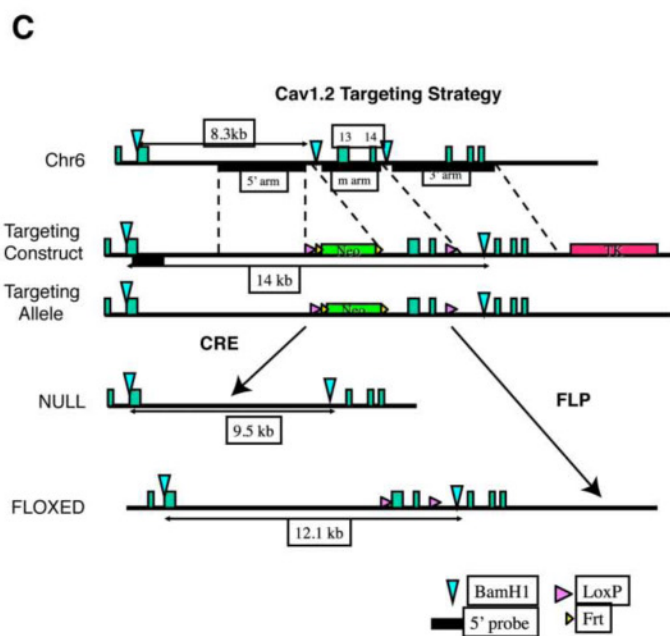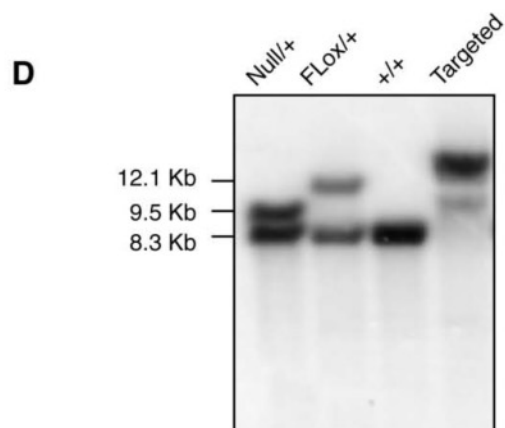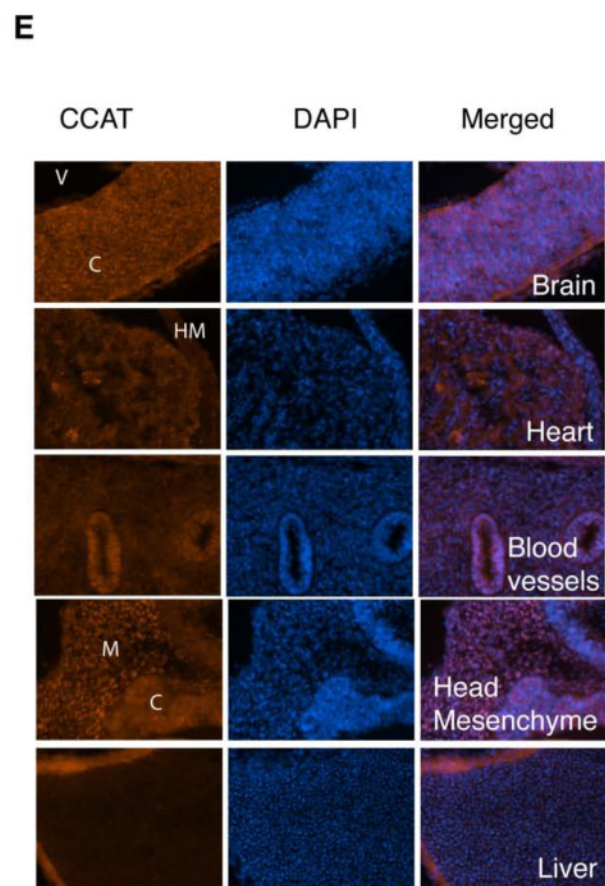

Supplement: Figure S4 — (A) Schematic representation of the 5′RACE approach to determine the TSS for the CCAT transcript generated from Cav1.2-Gal4. Briefly, two sequential phosphatase treatments are used to inactivate truncated or non-mRNAs and prepare intact, originally capped mRNAs for ligation of an RNA oligo to the uncapped 5′ end. In this experiment, reverse transcription was performed with a Gal4 reverse primer. Nested primers within the 5′ tag and the Gal4 coding sequence were used for PCR. The bands were then cloned and sequenced. (B) Agarose gel of PCR products amplified after performing 5′ RACE, as described in A, of Neuro2A cells expressing Cav1.2-Gal4 channels with and without the CMV promoter. (C) Schematic of the Cav1.2 knockout strategy. (D) Southern blot showing the efficacy of recombination and the expected molecular weight of the BamHI-digested genomic fragments after recombination. (E) Immunohistochemistry of heterozygote 11.5 dpc embryos stained with anti-CCAT antibody. Membranous staining is seen in the developing cortex and heart muscle wall (HM). Staining is noticeably nuclear in somites, mesenchymal cells and blood vessels. CCAT staining is not detected in the liver. (PDF) [file pone.0060526.s004.pdf]
