# Supplementary material for: A Promoter in the Coding Region of the Calcium Channel Gene CACNA1C Generates the Transcription Factor CCAT
Source: PLoS One. 2013 Apr 16;8(4):e60526. doi: 10.1371/journal.pone.0060526 (PMC3628902; doi:10.1371/journal.pone.0060526)
Supplement: Methods S1 — Description of reagents and antibodies used, plasmid generation, cell culture and transfection, luciferase assay transfection, Northern blots, single cell qPCR primers, 5′ RACE experiments, sequence analysis and multiple sequence alignments. (DOC) [file pone.0060526.s005.doc]

**Methods S1**

**Materials**

Brefeldin A was purchased from Calbiochem and used at 10µM. The generation of anti-CCAT was described earlier (Gomez-Ospina et al., 2006) and was used at 1:500-1:1000 for Western blots. Anti-gal4 SC-510, purchased from Santa Cruz Biotechnology, was used at 1:200 for Western blots and 4 µg for immunoprecipitations. ß-actin (1:2000) and CREB (clone NL904) (1:1000) antibodies were purchased from Ambion and Upstate/Millipore, respectively. Cultured neurons were stained primarily with rabbit anti-CCAT (1:500) and secondarily with Alexa 594-conjugated or Alexa 488-conjugated anti-rabbit antibodies (1:500; Molecular Probes). Nuclei were stained using Hoechst 33258 or DAPI (Molecular Probes).

**Plasmid Construction**

Note: All mutations were generated using the Quick Change XL mutagenesis kit, according to manufacturer’s instructions. All amino acid and base pair positions in Ca_v_1.2 refer to Acc# AAA18905.1.

Construction of the dihydropyridine-resistant Ca_v_1.2-Gal4 and ∆TA channels has been previously described (Dolmetsch et al., 2001; Gomez-Ospina et al., 2006). Construction of ∆TM-IQ was achieved in a three-step process. First, we inserted a KpnI site immediately downstream of the IQ at position 4929 of the Ca_v_1.2-Gal4 channel’s coding sequence, which was accomplished by PCR amplification with the following primers:

4930-4965 Fwd 5’-GGCAAGCCCTCGCAGAGGAATGCACTGTCTCTGCAG-3’ and IQ reverse (4893-4929) 5’-GGTACCGACCAGCCCCTGCTCTTTTCGCTTCTTGAATTTCCTG-3’.

The amplicon was a linearized channel vector with the KpnI site added to the reverse primer. The PCR product was then DpnI digested, blunt ligated and transformed.

The second step was to insert another KpnI site at the 4477-4482 nucleotide position, maintaining the channel’s reading frame. The primers used were: (4477-4482) KpnI fwd 5’-GGATTGGTCTATCCTTGGTACCCATCACCTGGATGAATTCAAGAG-3’

(4477-4482) KpnI rev 5’-CTCTTGAATTCATCCAGGTGATGGGTACCAAGGATAGACCAATCC-3’

Finally, to obtain ∆TM-IQ, the mutant plasmid was subjected to KpnI digestion, gel extraction, ligation and transformation.

The delta cleavage site channel was generated by deleting 150 AA downstream of the IQ motif. The Cav.12-Gal4 channel was amplified using the following primers: 5371-5406 Fwd GTCAGCACTGTGGAGGGCCATGGGCCTCCCTTGTCC

IQ reverse (4893-4929) GGTACCGACCAGCCCCTGCTCTTTTCGCTTCTTGAATTTCCTG.

This linear constructed was then DpnI treated, blunt ligated and transformed.

The translational stop channel was created after deletion of 193 bp between nucleotides 5588 and 5781 using the double KpnI site strategy with the following primers: 5588 KpnI fwd 5’-GCTCTCCACAGATATACTCTGGTACCAGGACGATGAAAACCG-3’

5588 KpnI rev 5’-CGGTTTTCATCGTCCTGGTACCAGAGTATATCTGTGGAGAGC-3’ and 5781 KpnI fwd 5’-GCCTTGCCCTTGCATCTGGTACCTCACCAGGCATTGG-3’

5781 KpnI rev 5’-CCAATGCCTGGTGAGGTACCAGATGCAAGGGCAAGGC-3’

After KpnI digestion and excision of the intervening sequence, there was a frameshift in the sequences, creating an early stop codon at AA. 1910.

Methionine mutations of Cav1.2-Gal4 channels and CCAT minigenes were generated using the primers:

M2011I: F 5’-TTGGCAGTGGCAGGGATCCCCCGGAGAGCCCGG-3’

R 5’-CCGGGCTCTCCGGGGGATCCCTGCCACTGCCAA-3’

(Primers add a silent Cspc site used for screening mutants).

M2073I: F 5’-CTGGCTGACGCCTGCGATATCACAATAGAGGAGATGGAG-3’

R 5’-CTCCATCTCCTCTATTGTGATATCGCAGGCGTCAGCCAG-3’

(Primers add a silent EcorV site used for screening mutants).

M2078I: F: 5’CGACATGACAATAGAGGATATCGAGAACGCCGCAGACAACATC-3’

R: 5’-GATGTTGTCTGCGGCGTTCTCGATATCCTCTATTGTCATGTCG-3’

(Primers add a silent EcorV site used for screening mutants).

Promoterless constructs were generated by MfeI digestion and self-ligation of the pcDNA4 Ca_v_1.2-Gal4 plasmid. This removed an additional 407 base pairs from the channel’s 5’ end.

The Ca_v_1.2 coding sequence as promoter constructs (Figure 2D and 2E), except for the full length channel sequence, were built by PCR amplification of Exons1-45 (1-5793 nt), E46-E47 (5794 – 6429 nt), E46 (5794-6129 nt) and E47 (6130-6429 nt) and insertion via KpnI digestion into the pGL3 basic luciferase reporter vector (Promega). The full length channel sequence was subcloned from PA1-Cav1.2 plasmid, which contains the full length channel’s sequence between two KpnI sites in the PA1 expression vector. This vector was originally obtained from Dr. Michael Lin and described earlier (Gomez-Ospina et al., 2006). The channel’s termination codon and an additional stop codon in between the channel’s and luciferases’s CDS were mutated using the following primers:
Pgl3- Ca_v_1.2 6490 stop

F 5’-GAGATCTGCGATCTAAGGAAGCTTGGCATTCCGGTACTG-3’

R 5’-CAGTACCGGAATGCCAAGCTTCCTTAGATCGCAGATCTC-3’

Pgl3- Cav1.2 6436 stop

F 5’-CTATGTCAGCAACCTGTACGGTACCGAGCTCTTACGC-3’

F 5’-GCGTAAGAGCTCGGTACCGTACAGGTTGCTGACATAG-3’

Minigenes were generated using a multistep cloning process: 1. An NcoI site was mutated to a MluI site in the pGL4.10 vector (Promega). 2. An amplified Zeocin resistance cassette from pCDNA4 was inserted into the BamI/Sal1 sites of pGL4.10. 3. To create WT, a 4 kilobase genomic region was PCR amplified using NcoI/MluI sites and the BAC clone RP23-158O9 3’ as a template. The Gal4-based minigene was generated by removing the luciferase and subcloning a PCR-amplified sequence of Gal4-DBD using the restriction sites MluI and Xba1. FS was created using the mutagenesis kit to insert a G between the last amino acid of the channel and the Gal4 coding sequence in the WT construct. ∆238 was created by inserting a CspcI site at 1579 bp of WT construct within exon 46. The sequence already had a CspcI site at the 5’ end of Exon 47. Subsequent digestion with CspcI removed a 238 bp sequence.

The plasmids encoding the N-terminal tagged YFP-mem-CCAT and YFP-CCAT were generated using Gateway technology (Invitrogen) by cloning the sequences from the TSS2 for mem-CCAT and TSS for CCAT amplified from a pCDNA4.0 Ca_v_1.2 plasmid into the TOPO sites of the pCR8 entry vector. The Ca_v_1.2 coding sequence was subsequently transferred into a destination vector called pDEST-pGWYFP that contains a CMV promoter and an N-terminal YFP in frame with the ATTR acceptor sequences.

**Cell Culture and Transfection**

Cortical and thalamic neurons were dissected from E17-19 Sprague Dawley rats in ice-cold Hank's Balanced Salt Solution without Ca++ and Mg++ (HBSS, Gibco). Thalami were enzymatically digested using trypsin (Worthington, 10mg/ml) and DNase (Sigma, 200 U/ml) in HBSS at room temperature for 10 min. Thalami were washed 3x in Basal Medium Eagle with 5% FBS, P/S, LQ and 1% glucose and gently triturated in the same media. Neurons were plated at 25,000/cm2. Arabinosylcytosine was added 24 h after plating to inhibit glial cell growth. Neuro2As (24 hr), cortical and thalamic neurons (96 hr) were transfected using lipofectamine 2000, according to manufacturer's instructions.

**Luciferase Assay Transfections**

Cav1.2-Gal4 channels were transfected in a ratio of 2:1:1:0.5 for Ca_v_1.2, the β1b subunit, the firefly luciferase and the Renilla luciferase reporters. Minigenes were transfected as a 1:1:0.25 ratio of minigene to UAS-firefly luciferase to Renilla luciferase. Channel coding sequence as promoter constructs were transfected at a ratio of 2:1, pGL3 based vector to Renilla luciferase.

**Northern Blots**

Probe: Exon 47-pGL3 plasmid was used as a template to PCR amplify the Exon 47 sequence. After gel extraction, 25ng of PCR template was used for labeling with the prime-it II random primers labeling kit (Stratagene) and 5µl of {α-32P]dCTP at 3000 Ci/mmol (Amersham), following the manufacturer’s protocol. For a loading control, a 900bp region of the 18S RNA was amplified and cloned using the following primers: 18S RNA L 5’-GAGGGAGCCTGAGAAACGGCTA-3’ 18S RNA R 5’-AACTAAGAACGGCCATGCACCA-3’ and used as a template for random labeling as described above. The full length channel probe contained sequence nt 2659 to 2966 in domain III of the channel’s coding sequence and was amplified using the following primers:

IIIS1 F 5’-GCGAAGCTTagcccaaacaacaggttc-3’ IIIS3 R 5’-gcgAAGCTTatgccaaaggagatgagg-3’

Briefly, 5µg of mRNA was loaded onto a 1% RNAse free agarose gel. Electrophoresis was carried out at ~5 V/cm. RNA was then transferred to Ambion’s BrightStar-Plus membranes by downward capillary transfer. The RNA was crosslinked to the membrane using a commericial crosslinker. Membranes were prehybridized at 68°C for 5-6h in prehybridization solution plus 100µg of salmon sperm DNA. Labeled probe was denatured before it was added to hybridization solution and incubated at 55-65°C overnight. Membranes were washed using low and high stringency washes as outlined in the Northern Max kit. Films were exposed for 5-48h at -80°C.

**Single Cell qPCR primers**

Mouse:

Exon 47-F               CCTAATGGGTTCGTTTCAGAAGT

Exon 47-R               TCCGGTTACCTCCAGGTCA

Human:

Exon 47-F            GCGACATGACCATAGAGGAGAT

Exon 47-R            CGTCCCTGCAGTTCACAAA

Exon 8a-F ACGCTATGGGCTATGAGTTACC

Exon 8a-R GGCCTTCTCCCTCTCTTTG

Exon 8-F TTTGACAACTTTGCCTTCGC

Exon 8-R TCCCTTCCTACGGCATCATT

Primer pair efficiencies:

Exon 8: 95%

Exon 8a: 110%

Exon 47: 102%

GSX-GSH2-F           ATGTCGCGCTCCTTCTATGTC

GSX-GSH2-R           ATGCCAAGCGGGATGAAGAAA

CMTM5-F            GGAGGACCACATCCGCTAGAT

CMTM5-R            CCAGGGAGTGGAAGCAGAT

DLX1-f                  CCATGCCAGAAAGTCTCAACA

DLX1-r                  GGCCCAAACTCCATAAACACC

FOXP1-F               AGACAAAAAGTAACGGTTCAGCC

FOXP1-R              CGCACTCTAGTAAGTGGTTGC

GAD67-F              GCCAGACAAGCAGTATGATGT

GAD67-R              CCAGTTCCAGGCATTTGTTGAT

NKX2.1-F             AGCACACGACTCCGTTCTC

NKX2.1-R             GCCCACTTTCTTGTAGCTTTCC

MSX2-F                CACCCTGAGGAAACACAAGAC

MSX2-R                AACTCTGCACGCTCTGCAAT

**5’ RACE experiments**

250ng of each mRNA template was dephosphorylated using calf intestinal phosphatase (CIP) at 50°C for 1h. After precipitation the cap structure was removed using tobacco acid pyrophosphatase (TAP) at 37° C for 1h. After precipitation, the RNA oligo 5’-CGACUGGAGCACGAGGACACUGACAUGGACUGAAGGAGUAGAAA-3’ was ligated to the 5’ end using T4 RNA ligase by incubating at 37C for 1 h. Reverse transcription followed using the RACE outer primer 5’-CTACAGGTTGCTGACATAGGACCTGCT-3’ encompassing sequence including the channel’s termination codon or olido-dT primer. PCR amplification was carried out using the primers: GeneRacerTM 5’-CGACTGGAGCACGAGGACACTGA-3’ primer and 5’RACE reverse inner primer 5’-CACAAAAGGTAAGAGGGTGCCGTTG-3’. To increase the abundance of longer transcripts, a reverse primer closer to the 5’ end (5970nt of cDNA) was used for CDNA synthesis (5’-GAAGCTGCTGTTGAGTTTCTCACTGGACTC-3’) and the nested 5'-CTGGTGATGAACCAGATGCAAGGGCA-3’ (5793 nt) was used for amplification.

GeneRacerTM 5′ Nested Primer 5’-GGACACTGACATGGACTGAAGGAGTA-3’

5’ RACE of the second transcript from the Cav1.2-Gal4 channel was accomplished using the following primers: for cDNA synthesis Cav1.2-Gal4 6865 (up to stop) R:

5’-TGACCGGCGATACAGTCAACTGTCTTTG-3’ and for PCR the Cav1.2-Gal4 6565 R

5’-TCAGCGGAGACCTTTTGGTTTTGGG-3’ and GeneRacerTM 5’ as the forward primer.

**Sequence Analysis and Multiple Sequence Alignments**

The following accession numbers were used for alignments:

**Ca_v_1:** *Drosophila Melanogaster* NP_602305.1, *Caenorhabditis elegans* NP_001023079.1

**Ca_v_1.2:** Human NP_001123312.1 Mouse NP_001153006.1 Zebrafish NP_571975.1

Rat [P22002.1](http://www.ncbi.nlm.nih.gov/entrez/query.fcgi?cmd=Retrieve&db=Protein&list_uids=116412&dopt=GenPept&RID=PMTFNFYM01N&log$=prottop&blast_rank=1), Chimpanzee XP_522315.2, Rhesus monkey XP_001117926.1, Guinea pig dbj|BAA34185.2, Rabbit NP_001129994.1, Horse XP_001490707.1, Dog XP_534932.2, Bovine XP_001255123.2, Chicken XP_416388.2

Sequences were aligned with ClustalW and MAFFT multiple sequence alignment programs. The alignments were edited using Jalview and colored using percentage identity with a conservation color increment set to 20.
